# Evaluation and optimization of a commercial enzyme linked immunosorbent assay for detection of *Chlamydophila pneumoniae *IgA antibodies

**DOI:** 10.1186/1471-2334-8-98

**Published:** 2008-07-26

**Authors:** Olfa Frikha-Gargouri, Radhouane Gdoura, Abir Znazen, Nozha Ben Arab, Jalel Gargouri, Mounir Ben Jemaa, Adnene Hammami

**Affiliations:** 1 Department of Microbiology and research laboratory "Microorganismes et Pathologie Humaine", Habib Bourguiba Hospital of Sfax, Tunisia; 2 Department of Infectious Diseases, Hedi Chaker Hospital of Sfax, Tunisia; 3 Department of Blood Bank, Sfax, Tunisia

## Abstract

**Background:**

Serologic diagnosis of *Chlamydophila pneumoniae *(Cpn) infection routinely involves assays for the presence of IgG and IgM antibodies to Cpn. Although IgA antibodies to Cpn have been found to be of interest in the diagnosis of chronic infections, their significance in serological diagnosis remains unclear. The microimmunofluorescence (MIF) test is the current method for the measurement of Cpn antibodies. While commercial enzyme linked immunosorbent assays (ELISA) have been developed, they have not been fully validated. We therefore evaluated and optimized a commercial ELISA kit, the SeroCP IgA test, for the detection of Cpn IgA antibodies.

**Methods:**

Serum samples from 94 patients with anti-Cpn IgG titers ≥ 256 (study group) and from 100 healthy blood donors (control group) were tested for the presence of IgA antibodies to Cpn, using our in-house MIF test and the SeroCP IgA test. Two graph receiver operating characteristic (TG-ROC) curves were created to optimize the cut off given by the manufacturer.

**Results:**

The MIF and SeroCP IgA tests detected Cpn IgA antibodies in 72% and 89%, respectively, of sera from the study group, and in 9% and 35%, respectively, of sera from the control group. Using the MIF test as the reference method and the cut-off value of the ELISA test specified by the manufacturer for seropositivity and negativity, the two tests correlated in 76% of the samples, with an agreement of Ƙ = 0.54. When we applied the optimized cut-off value using TG-ROC analysis, 1.65, we observed better concordance (86%) and agreement (0.72) between the MIF and SeroCP IgA tests.

**Conclusion:**

Use of TG-ROC analysis may help standardize and optimize ELISAs, which are simpler, more objective and less time consuming than the MIF test. Standardization and optimization of commercial ELISA kits may result in better performance.

## Background

*Chlamydophila pneumoniae *(Cpn) is a common cause of acute respiratory infections, primarily pneumonia, as well as other acute upper and lower respiratory tract infections such as bronchitis, sinusitis, otitis and pharyngitis. Cpn infection is associated with 5% to 20% of cases of community acquired pneumonia in adults and children [1,2]. To date, however, no totally satisfactory serological method has been developed for the diagnosis of Cpn infection. The U.S. Centers for Disease Control and Prevention (CDC) has recommended that the microimmunofluorescence (MIF) method be the reference serological test, despite the poor predictive value of a single high IgG titer [3].

Diagnosis of acute Cpn infection is based on paired serum samples obtained 4 to 8 weeks apart showing a 4-fold increase in IgG antibody titer, or on a single sample showing IgM antibody positivity. IgM antibodies appear earlier than IgG antibodies, making the former useful for the rapid diagnosis of acute Cpn infections. The significance of the presence of chlamydial IgA antibodies for serological diagnosis of infection is unclear. The persistence of these short lived [4] specific IgA antibodies may be a marker of persistent infection [5], and has been used in the definition of chronic Cpn infection [6-10]. Studies have demonstrated an association between specific anti-Cpn IgA antibodies and several chronic diseases, including chronic obstructive pulmonary disease [11], cardiovascular disease [12,13], chronic pharyngitis [14] and chronic upper and lower respiratory tract infections [15].

The reference method for the serological diagnosis of Cpn infections is the MIF test. This test, however, requires a highly experienced reader, has several important subjective components, can be difficult to interpret, and usually requires both an acute and convalescent specimen to demonstrate an increase in antibody titer. Furthermore, it lacks standardization [16]. Due to these drawbacks, several partially automated commercial enzyme linked immunosorbent assays (ELISA) have been developed. Compared with MIF assays, they are relatively simple to perform, less time consuming, more objective and easier to standardize. However, these commercial ELISAs have not been fully validated. They seem to be less specific but more sensitive than the MIF test [3]. We therefore evaluated and optimized a commercially available ELISA kit, the SeroCP IgA test, for anti-Cpn IgA antibodies and compared it with our in house MIF test. This study was not diagnostic, but rather an assay evaluation, since no convalescent-phase sera were used.

## Methods

### Sera

Serum samples were obtained from 94 patients referred to the Department of Infectious Diseases, Hedi Chaker Hospital of Sfax, Tunisia, between January 2002 and November 2004 who had anti-Cpn IgG titers ≥ 256 by our in house MIF assay (study group). Serum samples were also obtained from 100 healthy blood donors (90 men; mean age, 34 years; range, 19–56 years). All serologic assays were performed by the Laboratory of Microbiology in the University Hospital of Sfax, Tunisia. All subjects provided verbal informed consent, and the study protocol was approved by our ethics committee (Association d'Enregistrement et de Lutte Contre le Cancer du Sud Tunisien).

### Techniques

#### MIF test

Cpn species specific IgG and IgA antibodies were measured by our in house MIF test as described by Wang and Grayston [17] using as antigens purified elementary bodies of Cpn, IOL-207 strain, *Chlamydophila psittaci *Loth strain and *Chlamydia trachomatis *(Ct) L2 strain. These antigens were produced in yolk sac membranes of infected eggs. The sacs of uninfected eggs were used as negative control. The purified elementary bodies were not further treated (e.g. by removal of lipopolysaccharide). Slides were prepared as acetone fixed preparations of the purified antigens by experienced laboratory technicians capable of maintaining all conditions equal between test runs. The antigen densities for all experiments were guaranteed by an optimal concentration of elementary bodies. IgG antibody titers were determined from serial twofold dilutions of sera, starting at 1/16, and IgA antibody titers were determined at a single dilution (1:12). Prior to IgA testing, all sera were absorbed with rheumatoid factor absorbent (Dade Behring Marburg GmbH) to remove IgG and rheumatoid factor interactions according to the manufacturer's instructions. All MIF series included a positive and a negative control sample. The slides were incubated with primary antibody for 30 minutes in a moisture chamber at 37°C, washed twice for 5 minutes each with PBS and incubated with 1:300 fluorescein isothiocyanate (FITC) conjugated anti human immunoglobulin (Biorad) for 30 minutes. The mounting fluid for setting coverslips on the slides contained glycerol in PBS buffer. The next day, the slides were examined by two experienced and independent readers using a fluorescent microscope (Zeiss AxioStar Plus) with × 40 objective. In case of discordant readings, the slides were assessed by a third reader. Results were interpreted using the same microscope, by the same readers, at the same time. Only fluorescence with evenly distributed elementary bodies was scored as a positive reaction.

#### SeroCP-IgA test

The SeroCP™-IgA^® ^test (SeroCP) (Savyon Diagnostics Ltd, Germany) was used to test for the presence of IgA antibodies to Cpn, following the manufacturer's instructions. The IgA cut off value (COV) was calculated as twice the mean absorbance value at 450 nm (A_450_) of the two negative controls tested in each run. To normalize the results of different runs, the cut off index for each sample was calculated as sample A_450_/COV. The threshold index for a positive test was 1, as recommended by the manufacturer.

### Statistics

All data were collected using standardized forms and analyzed by Epi-Info version 6. To assess the agreement between MIF and the SeroCP-IgA test, we used Ƙ (nominal scale variables) [18]. Ƙ < 0.20 was defined as poor agreement, Ƙ = 0.21 to 0.40 as fair agreement; Ƙ = 0.41 to 0.60 as moderate agreement; Ƙ = 0.61 to 0.80 as good agreement; and Ƙ = 0.81 to 1.00 as very good agreement. Two-graph receiver operating characteristic analysis (TG-ROC) [19] was used to optimize the cut off index of the SeroCP-IgA test.

## Results

### Seroprevalence of Cpn IgA antibodies

Using the in house MIF and SeroCP-IgA tests, IgA antibodies to Cpn were detected in 72% (68/94) and 89% (84/94) of sera from the study group, respectively. Furthermore, Cpn IgA antibody positivity was correlated with increased Cpn IgG antibody titers (Table 1).

**Table 1:** Positivity of IgA antibodies in relation to MIF IgG antibodies titers in the study group

MIF IgG	256	512	1024	≥ 2048
antibodies titers	n = 62	n = 16	n = 7	n = 9
MIF IgA + (%)	41 (66)	12 (75)	6 (85)	9 (100)
ELISA IgA + (%)	57 (86)	15 (94)	7 (100)	9 (100)

In the control group, the seroprevalence of Cpn IgA antibodies using the in house MIF and SeroCP-IgA tests was 9% (9/100) and 35% (35/100), respectively. Sixty of the 100 healthy blood donors (60%) had IgG anti-Cpn titers above 1/16 using the MIF test, and five of these sera had titers above 1/256 and were positive for anti-Cpn IgA antibodies by MIF and ELISA. Of the other 95 sera, with IgG antibody titers <1/256, 4 were IgA positive by MIF and 30 by ELISA.

### Reproducibility of the ELISA test

**Figure 1:**
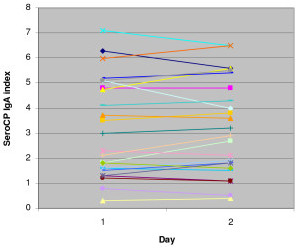
**Reproducibility of the SeroCP IgA ELISA assay**. ODs at 450 nm were obtained from two ELISA tests conducted on the same serum samples on two different days. The differences between OD 450 nm values were less than 20%.

Reproducibility of the ELISA test was determined by performing assays on the same serum samples, run under the same conditions, on different days. The differences between the OD values for sera with low, medium and high reactivity were each lower than 20% (Figure 1).

### Correlation between detection of Cpn IgA antibodies by MIF and ELISA before optimization

**Figure 2:**
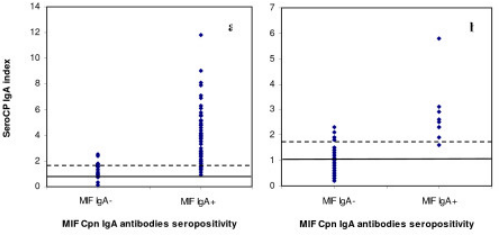
**Distribution of SeroCP IgA index in relation to MIF IgA antibody seropositivity**. **a**, Distribution of SeroCP IgA index in relation to MIF IgA antibody seropositivity in the study group. **b**, Distribution of SeroCP IgA index in relation to MIF IgA antibody seropositivity in the control group. The continuous lines are the manufacturer's cut off indices, and the discontinuous lines are the optimized cut off values.

Using the manufacturer's suggested cut off value, we observed a concordance of 76% and a moderate agreement (Ƙ = 0.54) between the MIF and SeroCP-IgA tests (Table 2, Figure 2). The concordance between the two tests was 78% for the study group and 74% for the control group (Table 2). Using the manufacturer's suggested cut off value, the agreement between the two tests was fair in both the study (Ƙ = 0.34) and control (Ƙ = 0.31) groups. Relative to the MIF test, the sensitivity and specificity of the SeroCP-IgA test before optimization were 97.4% and 62.4%, respectively (Table 3).

**Table 2:** Correlations between the detection of Cpn IgA antibodies by MIF and ELISA

	**Before optimization**			**After optimization**		
	
**MIF/ELISA**	**Total**	**Study ** **group**	**Control ** **group**	**Total**	**Study ** **group**	**Control ** **group**
	n = 194	n = 94	n = 100	n = 194	n = 94	n = 100
**+/+**	75	66	9	65	57	8
**-/-**	73	8	65	102	18	84
**+/-**	2	2	0	12	11	1
**-/+**	44	18	26	15	8	7
**Concordance (%)**	76	78	74	86	80	92
**Ƙ**	0.54	0.34	0.31	0.72	0.51	0.62

**Table 3:** Performance of the SeroCP IgA ELISA test before and after optimization

	**Before optimization**			**After optimization**		
	
**MIF/ELISA**	**Total**	**Study ** **group**	**Control ** **group**	**Total**	**Study ** **group**	**Control ** **group**
**Sensitivity (%)**	97.4	97.1	100	84.4	83.8	88.9
**Specificity (%)**	62.4	30.8	71.4	87.2	69.2	92.3
**PPV (%)**	63.0	78.6	25.7	81.3	87.7	53.3
**NPV (%)**	97.3	80.0	100	89.5	62.1	98.8

PPV: Positive predictive value, NPV: Negative predictive value

### Optimization of the SeroCP-IgA test

**Figure 3:**
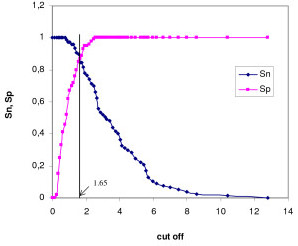
**TG-ROC analysis of the SeroCP-IgA test in sera from study and control groups**. The vertical line represents the suggested cut off value (1.65). Sn: sensitivity, Sp: specificity.

TG-ROC analysis was performed to optimize the cut off value for the SeroCP-IgA test. The sensitivity and specificity were plotted relative to the MIF results using different cut-off values (Figure 3), with the one having the highest sensitivity and specificity utilized. TG-ROC analysis showed that the optimal cut off index for the SeroCP-IgA test, relative to the MIF test, was 1.65.

### Correlation between detection of Cpn IgA antibodies by MIF and ELISA after optimization

Using the optimized cut off value, we observed a concordance of 86% and good agreement (Ƙ = 0.72) between the MIF and SeroCP-IgA tests (Table 2). The concordance was 80% in the study group and 92% in the control group, with moderate agreement (Ƙ = 0.51) between the MIF and SeroCP-IgA tests in the study group and good agreement (Ƙ = 0.62) in the control group. After optimization, the sensitivity and the specificity of the SeroCP-IgA test relative to the MIF test were 84.4% and 87.2%, respectively (Table 3).

## Discussion

We have compared the ability of our in house MIF assay and a commercial ELISA test to detect serum IgA antibodies to Cpn in patients suspected of having Cpn respiratory tract infections. Our MIF test was validated using reference sera and a commercial kit for Cpn (bioMerieux^®^, France) (data not shown). Using our MIF test, we found that the seroprevalence rates of Cpn IgA and IgG antibodies in healthy Tunisians were 9% and 60%, respectively, similar to findings in other studies, in which the seroprevalence rates of anti-Cpn IgA and IgG antibodies ranged from 5% to 50% and from 35% to 71%, respectively [1,10,12,20-26]. Thus, assays for IgG antibodies seem to be more sensitive for determining the seroprevalence of anti Cpn antibodies in a healthy population [25,26]. Using the MIF test, we observed a correlation between anti-Cpn IgA seropositivity and IgG titers, confirming previous results [27].

The performance of ELISA assays for anti-Cpn antibodies have been compared relative to the results of the MIF test, considered the standard method for the diagnosis of Cpn infections [25,28-30]. We focused on the SeroCP ELISA test, as its results more closely correlated with the results of MIF tests than did the results of other commercial diagnostic kits [31]. Our findings confirm that the SeroCP-IgA test was reproducible [32]. Before optimization, however, the correlation between the MIF and ELISA tests in detecting anti-Cpn IgA antibodies was not sufficient and the results of the two showed only moderate agreement. Our results are in agreement with those comparing the SeroCP-IgA test with a commercial MIF assay (Labsystems) [31]. In addition, we found that the SeroCP IgA ELISA test detected anti-Cpn IgA antibodies in samples that were negative by our in house MIF test. This finding agrees with results showing that the Labsystems IgA ELISA test also overestimated the prevalence of IgA antibodies (62%) compared with MIF (26%) [33]. Similar findings were reported for the detection of IgG antibodies by different serological methods [34].

The discrepancies between MIF and ELISA in the detection of anti-Cpn IgA antibodies may have been due to the different experimental conditions used in the two serological tests, including the Cpn strains, incubation times, antigen concentrations, serum dilutions, absorption experiments and the types of conjugates used.

Since both the SeroCP test and our MIF test use purified elementary bodies of Cpn as antigen, both assays measure antibodies directed against antigens localized on the surface of Cpn elementary bodies. However, the SeroCP test uses TWAR 183 as the reference strain, whereas our in house MIF uses the IOL-207 strain. Immunoblot analyses have detected antigenic differences between Cpn strains [35], and an MIF analysis of the antigenic profiles of six Cpn strains found that serum samples from culture positive patients had 4–8 fold higher titers against autologous strains than against TW-183 [36]. Furthermore, the criteria for establishing a diagnosis of acute infection were met only by the antigen from the local strain, not by TW-183. Thus, the serologic diagnosis of Cpn infection may require the use of antigens from more than one Cpn strain. In contrast, a comparison of the performance of three commercial MIF assays (WRF, Labsystems and MRL), which use different Cpn strains as antigens, found that their IgA endpoint titers were similar [37].

Increased incubation time and antigen concentration have been found to increase the sensitivity of the Labsystems MIF test to detect anti-Cpn IgA antibodies [38]. Our and commercial MIF tests have an incubation time of 30 minutes, whereas the SeroCP IgA test has an incubation of 1 hour. Furthermore, our in house MIF test uses an optimal concentration of elementary bodies, which was calibrated using the commercial MIF test and reference sera.

In addition, the SeroCP-IgA test used a higher dilution (1:105) than the MIF test (1:12), which may affect agreement between the methods [31]. This prozone effect with higher dilution of antibodies is supported by findings showing that the agreement between the MIF and ELISA tests was adequate at low but not at high titers and that the sensitivity of the ELISA test could be increased by testing sera with elevated titers at higher dilutions [39]. The manufacturer instructions for the SeroCP IgA test contain no data regarding the use of rheumatoid factor absorption. Absence of preabsorption experiment in the ELISA test may have increased the rate of false positive results, due to IgG interference.

One drawback to the measurement of anti-Cpn IgA antibodies may be the considerable variation in commercial anti-IgA conjugates, which may hamper the accurate detection of IgA antibodies [40,41]. In a comparative study of the IgA titers and seroconversions using six fluorescein anti-human IgA conjugates, only one of 14 seroconversions was detected by all 6 conjugates and in most, only one of the conjugates showed a significant increase in titer [40].

The discordance between MIF and ELISA may also be related to the increased sensitivity of the ELISA tests [26,33]. We found that the sensitivity of the SeroCP-IgA test, relative to our MIF test, was high before optimization (97.4%), but its specificity was limited (62.4%). Using the same antigen, we previously found that the specificity of the SeroCP IgG antibodies was 38.5% and that this test was not sufficiently specific to distinguish between IgG antibodies to Cpn and to Ct [29]. The MIF test is regarded internationally as the standard method for the determination of *Chlamydia *seropositivity [3]. Thus, the high sensitivity of ELISA tests could be interpreted in the reverse direction; rather, the specificity of the ELISA may be lower than that of the MIF test [42].

Although the MIF test is the currently recommended method for the measurement of Cpn antibodies [3] and is used in most clinical studies, this test is time consuming and requires skilled personnel for interpretation of the slides. Furthermore, its specificity has been questioned due to cross-reactions between chlamydial species [29,43]. Its ability to detect cross-reacting antibodies is not surprising because it detects antibodies against surface protein antigens of elementary bodies, which are shared by chlamydial species and other Gram negative bacteria [44-46]. In contrast to MIF, ELISAs are relatively simple, less time consuming, and more objective as they rely on photometric reading and are therefore easier to standardize. However, ELISA is relatively unreliable for Cpn antibodies detection compared with MIF [47]. Using ROC analysis in patients with coronary heart disease; the SeroCP-IgG test was found to correlate well against a commercial MIF assay (Labsystems), with the optimized cut off value corresponding to that recommended by the manufacturer [31]. In our study, we focused only on SeroCP-IgA antibodies as they have been found to generate a moderate concordance when compared to the MIF test [29,31]. Sera from healthy blood donors were used in our study in order to determine the prevalence of Cpn IgG and IgA antibodies and also to optimize the SeroCP IgA test. We found that only 9 out of these 100 sera were positive for IgA antibodies. Accurate optimization of the SeroCP IgA test required a homogeneous distribution of IgA antibodies in the serum samples. In addition, since our patients were clinically suspected of Cpn infection, we chose samples with IgG titers ≥ 256 instead of ≥ 512 to maximize the number of samples positive for IgA antibodies. When we used TG-ROC analysis to optimize the SeroCP IgA test in comparison to our MIF test, we found that test characteristics depended on the cut off value. Rather than using the cut off value recommended by the manufacturer, we estimated a new cut off value from the TG-ROC analysis to optimize the discrimination between positive and negative results [19]. TG-ROC analyses plot sensitivity and specificity as a function of cut off, with the cut-off value that optimized sensitivity and specificity utilized (1.65). Using this cut off index, we found a better concordance and agreement between the MIF and the SeroCP-IgA tests. If agreement between serologic tests is high, there should be no problems using either [34]. We found that, using our optimized cut off value, the sensitivity and specificity of the SeroCP-IgA test were 84.4% and 87.2%, respectively. Both sensitivity and specificity were higher in the control than in the study group, confirming results showing that the correlation between the MIF and SeroCP IgA tests was better with sera negative than positive for anti-Cpn IgA antibodies [39].

## Conclusion

The results of our study indicate that a high proportion of patients with anti-Cpn IgG titers ≥ 256 were positive for anti-Cpn IgA antibody. In comparing the SeroCP-IgA test with the MIF method, we found that the optimal cut off index for the SeroCP-IgA test was 1.65. TG-ROC analysis may provide an approach to the standardization and optimization of ELISAs, which are simpler, more objective and less time consuming than MIF tests. However, their difference in detecting anti-Cpn IgA antibodies suggests that the MIF continues to be the standard method for their measurement. Standardization and optimization of commercial ELISA tests, relative to the MIF test, may enhance the performance of the former.

## Competing interests

The authors declare that they have no competing interests.

## Authors' contributions

OFG performed laboratory experiments, evaluated and optimized the ELISA assays, analyzed data, and drafted the manuscript. RG and AZ participated in the analysis of data and coordination of the study. NBA participated in the collection of data. JG provided the sera from healthy donors and participated in the analysis of data. MBJ provided the patients' sera and participated in the analysis of data. AH participated in the experimental design, data analyses, and coordination of the manuscript and the study. All authors read and approved the final manuscript.

## Pre-publication history

The pre-publication history for this paper can be accessed here:



## References

[B1] Grayston JT, Kuo CC, Wang SP, Altman J (1986). A new *Chlamydia psittaci *strain, TWAR, isolated in acute respiratory tract infections. N Engl J Med.

[B2] Hahn DL (2000). *Chlamydia pneumoniae* antibodies and adult-onset asthma. J Allergy Clin Immunol.

[B3] Dowell SF, Peeling RW, Boman J, Carlone GM, Fields BS, Guarner J, Hammerschlag MR, Jackson LA, Kuo CC, Maass M, Messmer TO, Talkington DF, Tondella ML, Zaki SR, C. pneumoniae Workshop Participants (2001). Standardizing *Chlamydia pneumoniae* assays: recommendations from the Centers for Disease Control and Prevention (USA) and the Laboratory Centre for Disease Control (Canada). Clin Infect Dis.

[B4] Tomasi TB, Grey HM (1972). Structure and function of immunoglobulin A. Prog Allergy.

[B5] Saikku P (1999). Epidemiology of *Chlamydia pneumoniae* in atherosclerosis. Am Heart J.

[B6] Hahn DL, Anttila T, Saikku P (1996). Association of *Chlamydia pneumoniae* IgA antibodies with recently symptomatic asthma. Epidemiol Infect.

[B7] Saikku P, Leinonen M, Tenkanen L, Linnanmäki E, Ekman MR, Manninen V, Mänttäri M, Frick MH, Huttunen JK (1992). Chronic *Chlamydia pneumoniae* infection as a risk factor for coronary heart disease in the Helsinki Heart Study. Ann Intern Med.

[B8] Strachan DP, Carrington D, Mendall MA, Ballam L, Morris J, Butland BK, Sweetnam PM, Elwood PC (1999). Relation of *Chlamydia pneumoniae* serology to mortality and incidence of ischaemic heart disease over 13 years in the caerphilly prospective heart disease study. BMJ.

[B9] Toss H, Gnarpe J, Gnarpe H, Siegbahn A, Lindahl B, Wallentin L (1998). Increased fibrinogen levels are associated with persistent *Chlamydia pneumoniae* infection in unstable coronary artery disease. Eur Heart J.

[B10] von Hertzen L, Isoaho R, Leinonen M, Koskinen R, Laippala P, Töyrylä M, Kivelä SL, Saikku P (1996). *Chlamydia pneumoniae* antibodies in chronic obstructive pulmonary disease. Int J Epidemiol.

[B11] von Hertzen LC (1998). *Chlamydia pneumoniae* and its role in chronic obstructive pulmonary disease. Ann Med.

[B12] Campbell LA, Kuo CC, Grayston JT (1998). *Chlamydia pneumoniae* and cardiovascular disease. Emerg Infect Dis.

[B13] Saikku P, Leinonen M, Mattila K, Ekman MR, Nieminen MS, Mäkelä PH, Huttunen JK, Valtonen V (1988). Serological evidence of an association of a novel Chlamydia, TWAR, with chronic coronary heart disease and acute myocardial infarction. Lancet.

[B14] Falck G, Engstrand I, Gad A, Gnarpe J, Gnarpe H, Laurila A (1997). Demonstration of *Chlamydia pneumoniae* in patients with chronic pharyngitis. Scand J Infect Dis.

[B15] Falck G, Gnarpe J, Hansson LO, Svardsudd K, Gnarpe H (2002). Comparison of individuals with and without specific IgA antibodies to *Chlamydia pneumoniae*: respiratory morbidity and the metabolic syndrome. Chest.

[B16] Peeling RW, Wang SP, Grayston JT, Blasi F, Boman J, Clad A, Freidank H, Gaydos CA, Gnarpe J, Hagiwara T, Jones RB, Orfila J, Persson K, Puolakkainen M, Saikku P, Schachter J (2000). *Chlamydia pneumoniae* serology: interlaboratory variation in microimmunofluorescence assay results. J Infect Dis.

[B17] Wang SP, Grayston JT (1974). Human serology in *Chlamydia trachomatis* infection with microimmunofluorescence. J Infect Dis.

[B18] Landis JR, Koch GG (1977). An application of hierarchical kappa-type statistics in the assessment of majority agreement among multiple observers. Biometrics.

[B19] Greiner M, Sohr D, Gobel P (1995). A modified ROC analysis for the selection of cut-off values and the definition of intermediate results of serodiagnostic tests. J Immunol Methods.

[B20] Aldous MB, Grayston JT, Wang SP, Foy HM (1992). Seroepidemiology of *Chlamydia pneumoniae* TWAR infection in Seattle families, 1966–1979. J Infect Dis.

[B21] Gencay M, Dereli D, Ertem E, Serter D, Puolakkainen M, Saikku P, Boydak B, Dereli S, Ozbakkaloglu B, Yorgancioglu A, Tez E (1998). Prevalence of *Chlamydia pneumoniae* specific antibodies in different clinical situations and healthy subjects in Izmir, Turkey. Eur J Epidemiol.

[B22] Kuo CC, Jackson LA, Campbell LA, Grayston JT (1995). *Chlamydia pneumoniae* (TWAR). Clin Microbiol Rev.

[B23] Leinonen M (1993). Pathogenetic mechanisms and epidemiology of *Chlamydia pneumoniae*. Eur Heart J.

[B24] Saikku P (1992). The epidemiology and significance of *Chlamydia pneumoniae*. J Infect.

[B25] Hermann C, Graf K, Groh A, Straube E, Hartung T (2002). Comparison of eleven commercial tests for *Chlamydia pneumoniae*-specific immunoglobulin G in asymptomatic healthy individuals. J Clin Microbiol.

[B26] Romano Carratelli C, Nuzzo I, Cozzolino D, Bentivoglio C, Paolillo R, Rizzo A (2006). Relationship between *Chlamydia pneumoniae* infection, inflammatory markers, and coronary heart diseases. Int Immunopharmacol.

[B27] Vammen S, Lindholt JS, Andersen PL, Henneberg EW, Ostergaard L (2001). Antibodies against *Chlamydia pneumoniae* predict the need for elective surgical intervention on small abdominal aortic aneurysms. Eur J Vasc Endovasc Surg.

[B28] Persson K, Boman J (2000). Comparison of five serologic tests for diagnosis of acute infections by *Chlamydia pneumoniae*. Clin Diagn Lab Immunol.

[B29] Frikha-Gargouri O, Znazen A, Gdoura R, Gargouri B, Arab NB, Jemaa MB, Hammami A (2008). Usefulness of enzyme linked immunosorbent assays species specific in the detection of *Chlamydia trachomatis* and *Chlamydophila pneumoniae* IgG antibodies in patients with genital infections or respiratory tract infections. Pathol Biol.

[B30] Halvorsen DS, Borvik T, Njolstad I, Gutteberg TJ, Vorland LH, Hansen JB (2002). *Chlamydia pneumoniae* IgA- and IgG antibodies in young survivors of myocardial infarction. A comparison of antibody detection by a microimmunofluorescence test and an enzyme immunoassay. J Intern Med.

[B31] Ciervo A, Petrucca A, Visca P, Cassone A (2004). Evaluation and optimization of ELISA for detection of anti-*Chlamydophila pneumoniae* IgG and IgA in patients with coronary heart diseases. J Microbiol Methods.

[B32] Ngeh J, Gupta S, Goodbourn C (2004). The reproducibility of an enzyme-linked immunosorbent assay for detection of *Chlamydia pneumoniae*-specific antibodies. Clin Microbiol Infect.

[B33] Paldanius M, Bloigu A, Alho M, Leinonen M, Saikku P (2005). Prevalence and persistence of *Chlamydia pneumoniae* antibodies in healthy laboratory personnel in Finland. Clin Diagn Lab Immunol.

[B34] Hoymans VY, Bosmans JM, Van Renterghem L, Mak R, Ursi D, Wuyts F, Vrints CJ, Ieven M (2003). Importance of methodology in determination of *Chlamydia pneumoniae* seropositivity in healthy subjects and in patients with coronary atherosclerosis. J Clin Microbiol.

[B35] Jantos CA, Heck S, Roggendorf R, Sen-Gupta M, Hegemann JH (1997). Antigenic and molecular analyses of different *Chlamydia pneumoniae* strains. J Clin Microbiol.

[B36] Black CM, Johnson JE, Farshy CE, Brown TM, Berdal BP (1991). Antigenic variation among strains of *Chlamydia pneumoniae*. J Clin Microbiol.

[B37] Bennedsen M, Berthelsen L, Lind I, Infection, Atherosclerosis and Macrolide Antibiotics Group (2002). Performance of three microimmunofluorescence assays for detection of *Chlamydia pneumoniae* immunoglobulin M, G, and A antibodies. Clin Diagn Lab Immunol.

[B38] Gnarpe J, Sparr A, Nääs J, Lundbäck A (2000). Serological analysis of specific IgA to *Chlamydia pneumoniae*: increased sensitivity of IgA antibody detection using prolonged incubation and high antigen concentration. APMIS.

[B39] Vainas T, De Graaf R, Stassen FR, Kurvers HA, Grauls GE, Kitslaar PJ, Bruggeman CA (2003). *Chlamydia pneumoniae* serology: comparing a commercial enzyme immunoassay and microimmunofluorescence test in patients with cardiovascular disease. APMIS.

[B40] Paldanius M, Bloigu A, Leinonen M, Saikku P (2003). Measurement of *Chlamydia pneumoniae*-specific immunoglobulin A (IgA) antibodies by the microimmunofluorescence (MIF) method: comparison of seven fluorescein-labeled anti-human IgA conjugates in an in-house MIF test using one commercial MIF and one enzyme immunoassay kit. Clin Diagn Lab Immunol.

[B41] Wang S (2000). The microimmunofluorescence test for *Chlamydia pneumoniae* infection: technique and interpretation. J Infect Dis.

[B42] Yetkin G (2006). *Chlamydia pneumoniae* and coronary artery disease: controversial results of serological studies. Int Immunopharmacol.

[B43] Wong YK, Sueur JM, Fall CH, Orfila J, Ward ME (1999). The species specificity of the microimmunofluorescence antibody test and comparisons with a time resolved fluoroscopic immunoassay for measuring IgG antibodies against *Chlamydia pneumoniae*. J Clin Pathol.

[B44] Mitov I, Haralambieva I, Petrov D, Ivanova R, Kamarinchev B, Iankov I (2003). Cross-reactive monoclonal antibodies raised against the lipopolysaccharide antigen of *Salmonella* minnesota Re chemotype: diagnostic relevance. Diagn Microbiol Infect Dis.

[B45] Maurin M, Eb F, Etienne J, Raoult D (1997). Serological cross-reactions between *Bartonella* and *Chlamydia* species: implications for diagnosis. J Clin Microbiol.

[B46] Jackson LA, Cherry JD, Wang SP, Grayston JT (2000). Frequency of serological evidence of *Bordetella* infections and mixed infections with other respiratory pathogens in university students with cough illnesses. Clin Infect Dis.

[B47] Nishimura M, Hashimoto T, Kobayashi H, Fukuda T, Okino K, Yamamoto N, Mashida C, Kawagoe K, Fujita H, Inoue N, Takahashi H, Ono T (2005). Close association of *Chlamydia pneumoniae* IgA seropositivity by ELISA with the presence of coronary artery stenosis in haemodialysis patients. Nephrol Dial Transplant.

